# Polydactyly-Myopia Syndrome: Genetic and Ophthalmologic Perspectives

**DOI:** 10.7759/cureus.58235

**Published:** 2024-04-14

**Authors:** Uma Swaminathan, Sachin Daigavane, Nivesh Gupta

**Affiliations:** 1 Ophthalmology, Jawaharlal Nehru Medical College, Datta Meghe Institute of Higher Education & Research, Wardha, IND

**Keywords:** familial clustering, consanguinity, ophthalmologic examination, genetic syndrome, myopia, polydactyly

## Abstract

Polydactyly-myopia syndrome is a rare genetic condition characterized by the co-occurrence of polydactyly and myopia. Herein, we present the case of a 28-year-old Muslim male, born of consanguineous parents, who presented with complaints of diminished vision since childhood. Ophthalmologic examination revealed severe myopia with characteristic fundus changes indicative of high myopia. Additionally, the patient exhibited polydactyly in all limbs, with a positive family history of both polydactyly and myopia. This case underscores the importance of recognizing and managing rare syndromes to provide appropriate genetic counseling and clinical care. Further research is warranted to elucidate the underlying genetic mechanisms and optimize therapeutic strategies for polydactyly-myopia syndrome. Awareness of this syndrome among healthcare providers is essential to facilitate early diagnosis and intervention for affected individuals and their families.

## Introduction

Polydactyly-myopia syndrome is an exceptionally rare genetic disorder characterized by supernumerary digits and myopia. While both polydactyly and myopia are individually well-documented conditions, their co-occurrence in this syndrome presents a unique clinical challenge. The syndrome has been sporadically reported in the literature, with limited understanding of its etiology and pathogenesis. Polydactyly, defined as the presence of extra digits, can manifest in various forms, ranging from a simple duplication of a finger or toe to more complex presentations involving additional bones and joints. It is one of the most common congenital limb malformations, with an estimated prevalence of 1 in 1,000 live births [1]. The genetic basis of polydactyly is heterogeneous, with both autosomal dominant and recessive inheritance patterns described [2].

Myopia, or nearsightedness, is a common refractive error where distant objects appear blurred while close objects can be seen clearly. It is estimated to affect approximately 30% of the global population, making it one of the most prevalent ocular conditions worldwide [3]. Myopia is influenced by genetic and environmental factors, with genetic predisposition playing a significant role in its development [4]. The coexistence of polydactyly and myopia in polydactyly-myopia syndrome suggests a potential genetic link between the two conditions. However, the underlying genetic mechanisms remain poorly understood, and further research is needed to elucidate the genetic basis of this syndrome [5]. Familial clustering of cases has been reported, suggesting an inherited component, but no specific gene or genetic locus has been definitively associated with the syndrome [6].

Given the rarity of polydactyly-myopia syndrome, clinical management poses several challenges. Treatment typically involves surgical correction of polydactyly, when indicated, and refractive correction for myopia [7]. Ophthalmologic follow-up is crucial to monitor for complications associated with high myopia, such as retinal detachment and macular degeneration [8]. This case report aims to contribute to the literature on polydactyly-myopia syndrome by presenting a rare case and discussing its clinical features, management, and implications for genetic counseling.

## Case presentation

A 28-year-old Muslim male, born as the second issue of a third-degree consanguineous marriage, presented to the ophthalmology outpatient department with a longstanding complaint of diminished vision since childhood. The patient reported a history of wearing spectacles since the age of 21, with frequent changes in prescription noted throughout childhood. On initial examination, the patient’s visual acuity in the right eye was recorded as finger counting up to 1 meter, and in the left eye, finger counting up to 1 meter was also noted.

**Figure 1:**
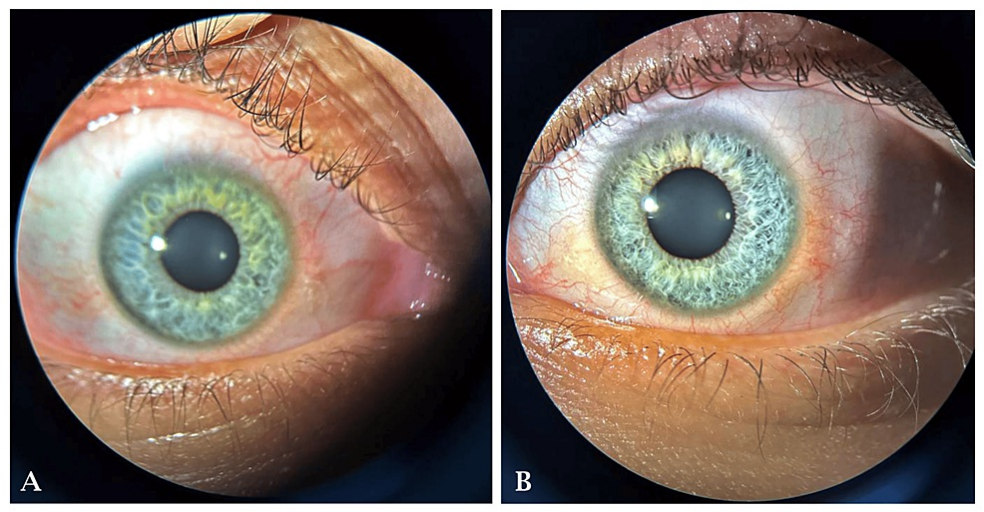
Slit lamp examination of the right and left eyes. A blue or green iris may indicate the carrier status of a hypomelanotic gene mutation.

Upon further assessment and subjective refraction, the best corrected visual acuity (BCVA) of the patient was determined to be 6/18 in the right eye, with a spherical equivalent of -17 diopters and a cylindrical equivalent of -2 diopters at an axis of 40 degrees. In the left eye, the BCVA was 6/12, with a spherical equivalent of -16 diopters and a cylindrical equivalent of -2 diopters at an axis of 150 degrees. Keratometry readings revealed corneal curvature with k1 = 43.37 and k2 = 44.25 in the right eye and k1 = 44.75 and k2 = 43.00 in the left eye. Axial length measured 29.72 mm in the right eye and 29.38 mm in the left eye. Intraocular pressure was within normal limits, measured at 19 mmHg on the right and 20 mmHg on the left. A slit lamp examination of the right and left eyes revealed a blue or green iris, indicating the carrier status of a hypomelanotic gene mutation (Figure 1).

**Figure 2:**
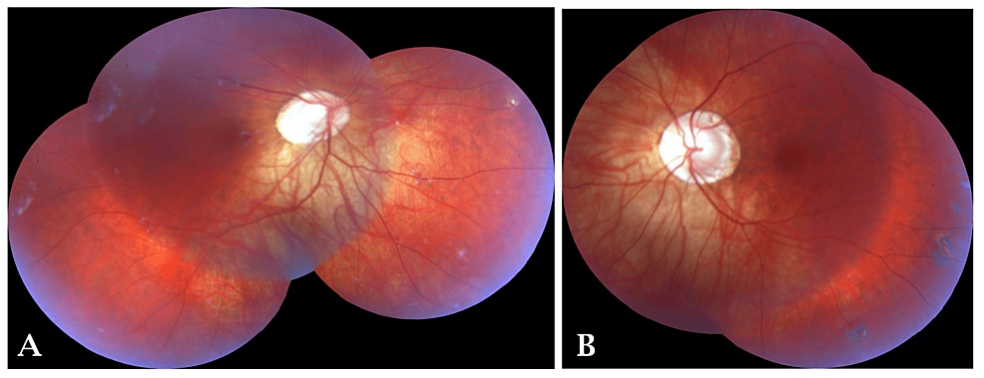
(A and B) Both eyes (OU) hypopigmented (albinotic) fundus with clear visualization of the arborizing network of choroidal vessels beneath the retina.

Fundus examination of both eyes revealed characteristic features of high myopia, including an albinotic myopic fundus with a large disc, peripapillary crescent, choroidal vessels, posterior staphyloma, and multiple chorio-atrophic scars present in the mid-periphery and equator. Despite the myopic changes, the foveal reflex was present bilaterally (Figure 2).

**Figure 3:**
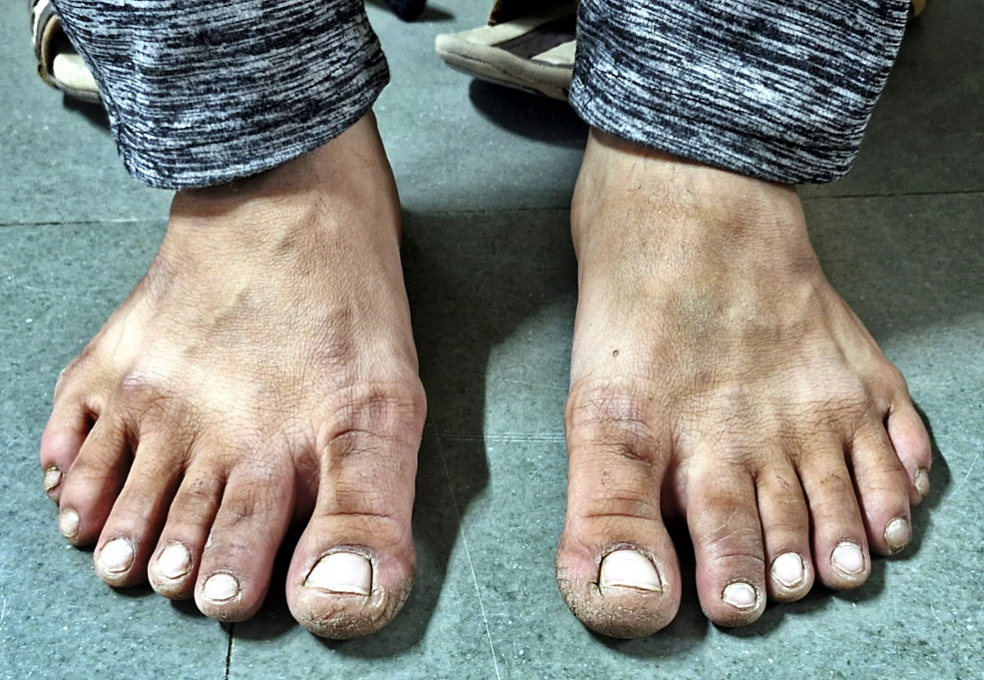
This shows fifth-digit duplicates in feet.

Physical examination revealed an additional anomaly: polydactyly in all limbs (Figure 3). The patient exhibited supernumerary digits in the hands, primarily composed of skin and soft tissue. Surgical intervention had been performed during childhood to address the polydactyly in the hands. Notably, the patient reported a positive family history of polydactyly, with affected individuals including the paternal grandfather, father, paternal uncle, and niece. Additionally, the patient’s mother and niece were reported to have myopia.

The constellation of findings in this case, including severe myopia, characteristic fundus changes consistent with high myopia, and polydactyly in the patient and his family members, suggests a diagnosis of polydactyly-myopia syndrome. This rare syndrome poses challenges in clinical management and genetic counseling, emphasizing the importance of a multidisciplinary approach in caring for affected individuals. Further genetic studies may provide insights into the underlying etiology of this syndrome and inform strategies for its management and prevention.

## Discussion

Polydactyly-myopia syndrome is extremely rare, with few cases reported in the literature. The simultaneous occurrence of polydactyly and myopia challenges understanding its etiology and management. The association between polydactyly and myopia has been sporadically reported, with limited understanding of the underlying genetic mechanisms. As observed in our case, the presence of polydactyly in multiple family members suggests a genetic basis for the syndrome. Consanguinity, as seen in our patient’s parents, may further contribute to the inheritance of autosomal recessive traits associated with the syndrome [5].

The pathogenesis of myopia involves a complex interplay of genetic and environmental factors. Several genetic loci have been implicated in myopia development, including genes involved in ocular growth and development [4]. While the specific genetic mutations underlying polydactyly-myopia syndrome remain unknown, it is plausible that mutations affecting both ocular and limb development pathways contribute to its manifestation. Ophthalmologic examination plays a crucial role in diagnosing and managing polydactyly-myopia syndrome. Severe myopia, as observed in our case, predisposes individuals to various ocular complications, including retinal detachment, macular degeneration, and glaucoma [9]. Regular monitoring for these complications is essential to prevent visual impairment and preserve ocular health.

Surgical intervention may be necessary to address polydactyly-associated functional and cosmetic concerns. However, the timing and extent of surgical intervention should be carefully considered, considering the patient’s age, functional impairment, and potential surgical risks [10]. Genetic counseling is paramount in families affected by polydactyly-myopia syndrome. Understanding the mode of inheritance, recurrence risks, and available management options can empower families to make informed decisions regarding family planning and medical management [11].

## Conclusions

Polydactyly-myopia syndrome represents a rare yet intriguing clinical entity characterized by the simultaneous occurrence of polydactyly and myopia. While the precise genetic underpinnings of this syndrome remain elusive, familial clustering and consanguinity suggest a genetic basis for its manifestation. Ophthalmologic evaluation is critical in diagnosis and management, with severe myopia predisposing individuals to sight-threatening complications. Surgical intervention may be necessary to address functional and cosmetic concerns associated with polydactyly. Genetic counseling is essential for affected families to understand the inheritance pattern, recurrence risks, and available management options. Despite its rarity, awareness of polydactyly-myopia syndrome is crucial among healthcare providers to facilitate early diagnosis, appropriate management, and genetic counseling for affected individuals and their families. Further research is warranted to elucidate the underlying genetic mechanisms and optimize therapeutic strategies for this complex syndrome.
